# Malabsorption of iron as a cause of iron deficiency anemia in postmenopausal women

**DOI:** 10.12669/pjms.312.6462

**Published:** 2015

**Authors:** Khansa Qamar, Muhammad Saboor, Fatima Qudsia, Shafi Muhammad Khosa, Moinuddin, Muhammad Usman

**Affiliations:** 1 Khansa Qamar, M.Phil. (Hematology). Baqai Institute of Hematology, Baqai Medical University, Karachi, Pakistan; 2 Dr. Muhammad Saboor, Ph.D, MT(ASCP^i^). Baqai Institute of Hematology, Baqai Medical University, Karachi, Pakistan; 3 Fatima Qudsia, M.Phil (Hematology). Baqai Institute of Hematology, Baqai Medical University, Karachi, Pakistan; 4 Dr. Shafi Muhammad Khosa, MBBS, DCP, M.Phil. Baqai Institute of Hematology, Baqai Medical University, Karachi, Pakistan; 5 Dr. Moinuddin FRCP (C), FRCP (E). Baqai Institute of Hematology, Baqai Medical University, Karachi, Pakistan; 6 Dr. Muhammad Usman, Ph.D (Hematology). Baqai Institute of Hematology, Baqai Medical University, Karachi, Pakistan

**Keywords:** Malabsorption, Iron deficiency anemia, Postmenopausal women

## Abstract

**Objective:**

Malabsorption is one of the causes of iron deficiency anemia in postmenopausal women. The main objective of this study was to access the frequency of malabsorption in iron deficient anemic postmenopausal women.

**Methods:**

A total of 123 postmenopausal women were enrolled in the study. Of these 123 women, 50 were included as ‘control group’ and 73 patients with comparable severity of anemia were the ‘patient group’. Two tablets of ferrous sulfate (200 mg/tablet) along with one tablet of vitamin C (500 mg) were given to all participants. Serum iron levels were determined on samples collected from all participants before and after the administration of ferrous sulfate. Difference between before and after serum iron levels of normal and patients were compared.

**Results:**

No change in serum iron between sample one and sample two represented malabsorption. Out of 73, 5 postmenopausal anemic patients showed no change in their serum iron level after the administration of ferrous sulfate. This study shows that frequency of malabsorption of iron in postmenopausal women is 6.8%.

**Conclusion:**

Malabsorption should be considered as a prevalent cause of iron deficiency anemia in postmenopausal women. It should be properly diagnosed and iron response should be monitored properly in postmenopausal women with IDA after oral iron therapy. If a postmenopausal woman does not show any response to oral iron therapy, she should be evaluated for iron loss (blood loss and/or malabsorption). Intravenous route should be used for the administration of iron in these patients.

## INTRODUCTION

Iron deficiency anemia (IDA) is the most common nutritional deficiency anemia in developing as well as developed countries.^1^-^3^ It results from decreased iron stores due to inadequate iron intake, poor absorption and increased iron demand and/or blood loss.^4^-^6^ Iron is essential for the production of erythrocytes; if its supply is inadequate, red cell production declines. This leads to the development of microcytic and hypochromic anemia.^3^,^7^ IDA rarely occurs in isolation. It usually associated with other conditions such as hookworm infestation, nutritional deficiency, malabsorption, hemoglobinopathies etc.^8^,^9^

However, it is estimated that approximately 1.6 billion people around the world i.e. nearly 25% of world population has anemia.^10^-^13^ It is estimated that approximately 50% of anemic individuals are afflicted with iron deficiency.^8^,^10^

Gastrointestinal tract is the site of absorption of essential micronutrients e.g. iron, cobalamine etc. Most of GIT diseases relate to malabsorption of these essential nutrients that subsequently lead to nutritional anemia. In some GIT disorders anemia occurs due to bleeding.^14^,^15^ Most common and frequent gastrointestinal diseases that cause anemia include celiac disease, inflammatory bowel disease (IBD), tropical sprue, intestinal tuberculosis, cystic fibrosis and H. pylori infection.^14^-^16^

The most common cause of IDA in postmenopausal women is chronic gastrointestinal bleeding and/or malabsorption.^17^,^18^ Literature related to IDA in postmenopausal women is very scanty and needs attention to workup on this issue.

Purpose of this study was to assess malabsorption of iron as a cause of iron deficiency anemia in postmenopausal women. A clear documentation of this risk factor among this group of patients will help plan more effective strategies for the control of this nutritional disorder.

## METHODS

A total of 123 subjects i.e. 50 normal and 73 iron deficiency anemic postmenopausal women were enrolled in this study. Informed written consent was taken from all subjects. Selection criteria included; normal healthy and iron deficient postmenopausal women with no history of recent illness, bleeding and drug intake particularly iron supplements during the past two weeks. This study was approved by the ethics committee of Baqai Medical University, Karachi.

Blood samples were collected in vacutainers (BD) from all enrolled individuals; 3ml in EDTA tube for CBC and 4 ml in gel tube for determination of iron profile. Two tablets of ferrous sulfate (200 mg / tablet) along with one tablet of vitamin C (500 mg) were given to all individuals (normal as well as patients). Serum iron levels were determined on samples taken from all individuals before and after the administration of ferrous sulfate to evaluate the absorption of iron. First blood sample was collected after overnight fasting while second sample was collected after three hours of ferrous sulfate administration.

### Complete blood counts (CBC)

CBC of all samples were determined using automated cell analyzer (SysmexKx 21 Tokyo, Japan). This included Hb estimation, red cell count, white cell count, platelet count, packed cell volume (PCV), mean cell volume (MCV), mean cell hemoglobin (MCH), mean cell hemoglobin concentration (MCHC) and red cell distribution width (RDW).

### Serum iron

Serum iron levels of all samples were determined using micro lab 200.

### Serum ferritin

Serum Ferritin of all samples were measured using semi-automated ELISA STAT FAX-2100.

### Statistical analysis

Results were analyzed using Statistical Package for Social Sciences version 17 (SPSS) Chicago, IL, USA. Descriptive statistics was used for mean, SD and frequency. One-way ANOVA was applied for the comparison of groups. *p* value of <0.005 was considered statistically significant.

## RESULTS

Based upon the inclusion criteria of this study and laboratory parameters, all recruited individuals (N= 123) were divided into two groups i.e. normal postmenopausal women (N= 50) and iron deficient postmenopausal women (N= 73). Table-I summarizes the results of CBC and iron profile of all subjects (*P* <0.001) i.e. normal IDA.

**Table-I:** CBC and serum iron profile of normal and iron deficient anemic postmenopausal women.

Parameters	Normal(N=50)	IDA(N=73)	p value
Hemoglobing/dl	11.93 ± 0.860	7.23 ± 1.760	0.001
Red cell countM/μl	4.53 ± 1.15	3.10 ± 0.93	0.001
MCVfl	80.36 ± 9.41	69.49 ± 8.68	0.001
MCHpg	26.16 ± 3.67	19.90 ± 3.10	0.001
MCHC%	31.08 ± 1.56	27.08 ± 2.16	0.001
RDW%	12.91+1.7	22.3+3.9	0.001
Serum iron 1μg/dl	117.06 ± 23.22	50.11± 35.76	0.001
Serum iron 2μg/dl	228.16 ± 66.78	185.47 ± 67.84	0.001
TIBC μg/dl	328.78 ± 43.15	652.27 ± 172.58	0.001
% Sat	35.94 ± 7.19	7.89 ± 4.66	0.001
Ferritinng/ml	81.92 ± 23.24	22.86 ± 16.86	0.001

Data are shown as mean ± SD. Hb= Hemoglobin, MCV= Mean Cell Volume, MCH= Mean Cell Hemoglobin MCHC= Mean Cell Hemoglobin Concentration Serum iron 1= serum iron before administration of ferrous sulfate, Serum iron 2= serum iron after administration of ferrous sulfate, TIBC= Total Iron Binding Capacity, % sat= % saturation of transferrin.

Levels of serum iron 1and ferritin in iron deficient females showed a wide range that fell into near normal range. Data were re-evaluated and it was found that there are 14 subjects with a normal level of serum iron 1 and serum ferritin levels. These individuals were sub grouped as comorbid iron deficiency anemia with chronic disorder (ACD+ID). Table-II summarizes the results of CBC and serum iron profile of iron deficient anemic females i.e. pure IDA and ACD+ID.

**Table-II:** CBC and serum iron profile of pure iron deficient anemic and comorbid iron deficiency anemia with chronic disorders.

Parameters	Pure IDA(N=59)	ACD+ID(N=14)	p value
Hemoglobing/dl	7.08 ± 1.72	7.82 ± 1.84	0.161
Red cell countM/μl	3.06 ± 0.93	3.23 ± 0.97	0.550
MCVfl	68.98 ± 8.82	71.64 ± 7.99	0.306
MCHpg	19.79 ± 3.28	20.36 ± 2.24	0.546
MCHC%	26.96 ± 2.22	27.57 ± 1.86	0.346
RDW %	22.3+3.9	19.2 ±1.9	0.079
Serum iron 1μg/dl	36.10 ± 8.39	109.14 ± 46.12	0.001
TIBC μg/dl	649.36 ± 143.84	664.64 ± 269.17	0.840
% sat	5.82 ±1.87	16.58 ± 2.059	0.001
Ferritinng/ml	6.88 ± 3.34	58.96 ± 22.02	0.001

Data are shown as Mean ± SD. Hb= Hemoglobin, MCV= Mean Cell Volume, MCH= Mean Cell Hemoglobin, MCHC= Mean Cell Hemoglobin Concentration, Serum iron 1= serum iron before administration of ferrous sulfate, TIBC= Total Iron Binding Capacity, % sat= % saturation of transferrin.

To determine the prevalence of malabsorption, levels of serum iron 1 were compared with those of serum iron 2. There were 5 anemic patients who showed no change in their serum iron level after the administration of ferrous sulfate. Frequency of malabsorption is shown in Table-III.

**Table-III:** Frequency of malabsorption and normal absorption in postmenopausal anemic patients.

Frequency	N	%
Malabsorption	05	6.8
Normal absorption	68	93.2

## DISCUSSION

Malabsorption is the inability of gastrointestinal tract to absorb ingested nutrients and micronutrients. Various diseases such as celiac disease, inflamatory bowel disease (IBD), whipple’s disease, tropical sprue etc. may lead to malabsorption of essential nutrients i.e. iron, vitamin B_12_, folate and vitamin A.^19^,^20^ Malabsorption diseases such as celiac disease, IBD and H.pylori gastritis are frequently associated with iron deficiency anemia.^7^,^21^,^22^

This study was undertaken with the primary object of determining the frequency of malabsorption in postmenopausal women. It was postulated that malabsorption may be a cause of iron deficiency anemia in postmenopausal women.

Interpretation of results was based on the change in the serum iron of the second sample i.e. after administration of oral iron. No change in serum iron between sample one and sample two represented malabsorption. It was found that second serum iron levels were increased in all individuals as compared with the first serum iron levels except in five iron deficient anemic postmenopausal women. This study shows that the frequency of malabsorption in postmenopausal women is 6.8%.

Goddard et al. have reported 5-10% prevalence of malabsorption in iron deficiency anemia. The commonest disease responsible for this was celiac disease.^17^ Vannella et al. studied the prevalence of bleeding and/or iron malabsorption associated diseases as a cause of IDA in elderly patients. They reported that gastrointestinal tract IDA related bleeding lesions were more frequent in elderly patients than in younger patients.^23^ Findings of Vannella et al. are at variance with our study. They reported that the prevalence of disease associated with malabsorption of iron is higher as compared with our study and it was more frequent in younger age group than in the elderly.^23^

Iron is absorbed in the proximal part of small intestine. Its absorption depends on various factors; intact mucosal surface being one of them. In gastrointestinal disorders, absorption of micronutrients like iron is impaired as a result of villous atrophy of small intestinal mucosa, malignancy of gastrointestinal tract, inflammation and bacterial overgrowth with consequent anemia. Collateral finding of our study showed significant number (19%) of iron deficient anemic postmenopausal women associated with comorbid anemia of chronic disorders (14/73) as shown in Table-II. Patients with ACD+ID had transferrin saturation <20% and serum ferritin between 30-100 ng/ml.

A number of workers have reported ACD with co-existing ID.^14^,^25^-^28^ Characteristics of this condition areincreased CRP, low transferrin saturation (<20%), normal serum ferritin (30-100 ng/ml) and increased sTfR/log ferritin ratio (>2). Pasricha et al. also found that ID associated with ACD is frequently seen in elderly patients.^14^

In ACD, cytokines such as TNF-α, IFN-γ, IL-6 etc. are produced as a result of activation of immune system. These inflammatory cytokines directly impair erythroid progenitor (BFU-E) proliferation, decrease red cell life span, impair the function of EPO and causes disturbance in iron homeostasis.^25^,^27^,^28^

Impaired iron hemeostasis develops due to increased expression of divalent metal transporter (IFN- γ) and transferrin receptor (IL-10) in macrophages, which increases the uptake of iron into macrophages. IFN-γ andIL-6 induced increased concentration of hepcidin in enterocytes down regulate the expression of ferroportin by blocking the release of iron from macrophages and inhibition of intestinal iron absorption. At the same time, TNF-α, IL-1, IL-6 and IL-10 increases the synthesis of ferritin in the macrophages (increased iron stores). Erythroid progenitor proliferation is impaired in ACD due to inhibitory factors such as TNF-α, IFN-γ, IL-1 (down regulator of erythropoiesis). While increased erythrophagocytosis occurs due to TNF-α and cytokine mediated toxic free radicals (nitric oxide, superoxide anion). Impaired EPO response also occurs due to inhibitory effect of IL-1 and TNF-α on EPO.^25^,^27^ Association of IDA and ACD is difficult to define; one assumption is that persistently decreased iron absorption due to increased hepcidin levels causes inhibition of intestinal iron absorption and prevent recirculation of iron in the macrophages. Chronic blood loss in ACD may precipitatetrue ID (ID+ACD).^25^,^27^,^28^

Limitations of this study include gastroscopy, small bowel enteroscopy and colonoscopy that were not done in our patients to identify the gastrointestinal tract diseases. C-reactive protein, EPO and sTfR were also not determined to exclude inflammation.

Oral iron supplementation is not the treatment of choice for these patients. Iron response should be monitored properly in postmenopausal women with IDA after oral iron therapy. For this purpose complete blood and reticulocyte count should be carried out on regular basis. This will help to monitor the iron response and evaluate refractory anemia. For the treatment of refractory anemia, thorough examination of gastrointestinal tract is required especially in postmenopausal women. If a patient does not respond to oral iron therapy, she should be referred to gastroenterology department for proper evaluation of iron loss (blood loss and/ormalabsorption) in postmenopausal women.

Intravenous iron administration is recommended for anemia caused by malabsorption. Treated patients should be regularly followed to evaluate the therapeutic response. Blood samples should be taken on a regular basis to check CBC, reticulocyte count as well as liver function test, serum iron, ferritin and transferrin saturation. Therapy should be stopped if any abnormality is seen in any of these parameters.

## References

[1] Nils M (2011). Anemia—still a major health problem in many parts of the world. Ann Hematol.

[2] Clark SF (2009). Iron deficiency anemia: diagnosis and management. Curr Opin Gastroenterol.

[3] Jeffery LM (2013). Iron deficiency anemia: a common and curable disease. Cold Spring Harb Perspect Med.

[4] Gnana-Prakasam JP, Martin PM, Smith SB, Ganapathy V (2010). Expression and function of iron-regulatory proteins in retina. IUBMB Life.

[5] Iqbal MS, Ahmed MS, Ogras TT, Ullah S, Asif JH, Keshavarzi F (2012). Diagnosis & management of iron deficiency anemia via parental Iron. Int J Nat Sci.

[6] Wang J, Pantopoulos K (2011). Regulation of cellular iron metabolism. Biochem J.

[7] Bermejo F, García-López S (2009). A guide to diagnosis of iron deficiency and iron deficiency anemia in digestive diseases. WJG.

[8] Shrivastava SR, Shrivastava PS, Ramasamy J (2013). Nutritional Anemia: Analysis of the existing gaps and proposed public health measures. Health Care.

[9] McLean E, Cogswell M, Egli I, Wojdyla D, de Benoist B (2009). Worldwide prevalence of anaemia, WHO vitamin and mineral nutrition information system, 1993–2005. Public Health Nutr.

[10] Balarajan Y, Ramakrishnan U, Ozaltin E, Shankar AH, Subramanian SV (2012). Anaemia in low-income and middle-income countries. Lancet.

[11] Pasricha SR, Drakesmith H, Black J, Hipgrave D, Biggs BA (2013). Control of iron deficiency anemia in low-and middle-income countries. Blood.

[12] Dolai TK, Nataraj KS, Sinha N, Mishra S, Bhattacharya M, Ghosh MK (2012). Prevalence of iron deficiency in thalassemia minor: A study from tertiary hospital. Indian J Hematol Blood Transfus.

[13] Lynch SR (2011). Why nutritional iron deficiency persists as a worldwide problem. J Nutr.

[14] Pasricha SR, Flecknoe-Brown SC, Allen KJ, Gibson PR, McMahon LP, Olynyk JK (2010). Diagnosis and management of iron deficiency anaemia: a clinical update. Med J Aust.

[15] Bayraktar UD, Bayraktar S (2010). Treatment of iron deficiency anemia associated with gastrointestinal tract diseases. World J Gastroenterol.

[16] Yadav P, Das P, Mirdha BR, Gupta SD, Bhatnagar S, Pandey RM (2011). Current spectrum of malabsorption syndrome in adults in India. Indian J Gastroenterol.

[17] Goddard AF, James MW, McIntyre AS, Scott BB (2011). Guidelines for the management of iron deficiency anaemia. Gut.

[18] Amy Z, Kaneshiro M, Kaunitz JD (2010). Evaluation and treatment of iron deficiency anemia: a gastroenterological perspective. Dig Dis Sci.

[19] Renee B (2011). Malabsorption: causes, consequences, diagnosis and treatment. S Afr J Clin Nutr.

[20] Vaníčková Z, Kocna P, Zima T (2012). Screening and confirmation of malabsorption. New Trends in classification, monitoring and management of gastrointestinal diseases.

[21] Owens SR, Greenson JK (2007). The pathology of malabsorption: current concepts. Histopathol.

[22] Fernández-Bañares F, Monzón H, Forné M (2009). A short review of malabsorption and anemia. World J Gastroenterol.

[23] Vannella L, Aloe Spiriti MA, Di Giulio E, Lahner E, Corleto VD, Monarca B (2010). Upper and lower gastrointestinal causes of iron deficiency anemia in elderly compared with adult outpatients. Minerva Gastroenterol Dietol.

[24] Bhasin A, Rao MY (2011). Characteristics of anemia in elderly: a hospital based study in South India. Indian J Hematol Blood Transfus.

[25] Muñoz M, Villar I, García-Erce JA (2009). An update on iron physiology. World J Gastroenterol.

[26] Theurl I, Aigner E, Theurl M, Nairz M, Seifert M, Schroll A, Sonnweber T (2009). Regulation of iron homeostasis in anemia of chronic disease and iron deficiency anemia: diagnostic and therapeutic implications. Blood.

[27] Weiss G, Goodnough LT (2005). Anemia of chronic disease. N Engl J Med.

[28] Bergamaschi G, Sabatino AD, Albertini R, Ardizzone S, Biancheri P, Bonetti E (2010). Prevalence and pathogenesis of anemia in inflammatory bowel disease;Influence of anti-tumor necrosis factor-α treatment. Haematologica.

